# Clinical Society of Maryland

**Published:** 1895-02

**Authors:** 


### *SOCIETY REPORTS.*

---

### CLINICAL SOCIETY OF MARYLAND.

BALTIMORE, December 7, 1894.

DR. WILLIAM BRINTON read a paper on "The Induction of Labor in Nephritis." (See page 719.)

DISCUSSION.

DR. MICHAEL: This question calls always for quick action, and delay is dangerous. I wish to say a word about the diagnosis. It is made often by the ophthalmologist. A doctor should make the examination of the kidney lesion himself, and it should be so well known to the obstetrician that he should not let the patient go to blindness. I should feel shabby if an ophthalmologist had to tell me of the existence of the disease. As to the treatment: I disapprove of Dr. Brinton's method of producing labor—that of using the bougie when a woman is having convulsions. Rapid dilatation by the finger is the safest and best method of bringing it on, though it is a difficult and troublesome plan. When the hand is used you run no risk of getting into the wrong place or doing any damage. The two remedies I like best are morphia and venesection. I do not know what venesection does except bring out a lot of bad blood, but it most surely produces good results. I believe that morphia and venesection are both extremely satisfactory, and that the latter has not been properly tried.

DR. WILLIAM T. HOWARD is a strong advocate of it. Before coming to Baltimore he had treated seven cases by free bleeding, and saved them all. The next six cases he saw here were treated differently, and all died. The next one was bled and got well. I believe the results are better on the average than are to be obtained in any other way.

DR. HIRAM WOODS: The question of the eye-symptoms is apt to be misunderstood, unless you bear in mind that there are two varieties of blindness associated with albuminuric conditions in pregnancy. One is the sudden failure of sight, such as described by Dr. Brinton, where there is no retinal lesion ; the other a case of true inflammation, with white plaques and decided retinal changes. The question is, whether in any of Dr. Brinton's cases there was true albuminuric retinitis. There were no ophthalmoscopic examinations made, and in all he said the blindness was sudden, and, with one exception, all got well. I can recall a patient in my care who had albuminuric retinitis in her first pregnancy, and her sight was reduced to a very small point. I followed her through four or five pregnancies, and although nearly blind in each, her sight was always restored to the point it had been left during the first pregnancy. Four of Dr. Randolph's cases had these changes, the other did not. The first case of his, which was referred to by Dr. Brinton, was not properly diagnosed. With a woman in her first pregnancy, with ensuing albuminuric retinitis, the question suggests itself, is premature labor in the fourth or fifth month justifiable? I should think it was, but how would that be regarded from an obstetrical point of view?

DR. TODD: I find that in New York the custom among the physicians is to justify the operation for the saving of life, but not simply to save eyesight.

DR. NORMENT: I wish to mention two cases seen recently—one in her fourth pregnancy. In her first she had eclampsia five or six weeks prior to labor, and conservative treatment was adopted. She was delivered of a child which had evidently been dead for some time. In her second, she had eclampsia during labor, and was delivered by forceps of a living child. In her third, she had a perfectly normal pregnancy and labor. In the fourth, I was sent for and found her in eclampsia in the eighth month of pregnancy. She was very large, weighing two hundred and forty pounds. There was no evidence of the onset of labor, and the difficulties of inducing labor, the condition of the patient, and the fact that she had been through the thing before successfully led us temporarily to postpone the induction of labor. We followed Dr. Michael's plan and bled her freely. She was stone blind, and I found any number of white plaques in the retina. Five weeks later she was delivered of a still-born child. There was little return of vision until after labor, but later it came up to about one-third normal.

*Case* 2.—I found a woman eight months pregnant in eclampsia for several hours, recognizing no one, and complaining of pain in the head. I bled her freely, she became conscious at once, and was altogether better. She had been perfectly blind, but soon was well enough to read the newspaper. She was afterwards delivered of a dead child. She had, I think, uremia without retinitis. I found no albumin in the urine.

Dr. Brinton: When we have time, certain methods can be used for inducing labor, but when in a hurry, the use of the finger is best. We did use morphia in one case, and with good results. I once reported four cases in which I had bled, and three recovered. In the next three, treated in the same manner, all died.

---

### Sugar as an Oxytocic.

On the strength of observations by Dr. Mosso and Dr. Paoletti as to the action of sugar on muscular power, Dr. Bossi conceived the idea of administering it in cases of defective uterine contraction during labor. He found that it answered the purpose well and was free from the inconveniences attending the action of ergot. In eleven cases of uterine inertia during labor an ounce of sugar dissolved in water was given, and in ten of the patients it had a most favorable effect on the pains.

The ecbolic action of sugar is apparent in from twenty-five to forty-five minutes, and in many cases it is sufficiently prolonged to accomplish the expulsion of the child. In some cases it has been found necessary to give a second dose of the same amount, an hour after the first one, in order to terminate the labor. The contractions excited by sugar are always perfectly regular, and never take on a tetanic character.—*Annals of Gynecology.*

